# A comprehensive assessment of the intertidal biodiversity along the Portuguese coast in the early 2000s

**DOI:** 10.3897/BDJ.9.e72961

**Published:** 2021-10-08

**Authors:** Joana Pereira, Pedro A Ribeiro, António Múrias Santos, Cátia Monteiro, Rui Seabra, Fernando P. Lima

**Affiliations:** 1 CIBIO/InBIO, University of Porto, Porto, Portugal CIBIO/InBIO, University of Porto Porto Portugal; 2 Department of Biological Sciences, University of Bergen, Bergen, Norway Department of Biological Sciences, University of Bergen Bergen Norway; 3 University of Porto, Porto, Portugal University of Porto Porto Portugal

**Keywords:** intertidal, biodiversity, rocky shores, historical data, Portugal

## Abstract

**Background:**

The unprecedented rates of current biodiversity loss have motivated a renewed interest in environmental and biodiversity monitoring. The need for sustained monitoring strategies has prompted not only the establisment of new long-term monitoring programmes, but also the rescue of data from historical or otherwise archived sources. Amongst the most valuable datasets are those containing information on intertidal systems, as they are particularly well suited for studying the biological effects of climate change. The Portuguese rocky coast is quite interesting for studying the effects of climate change on the distribution of species due to its geographical orientation, latitudinal patterns in temperature, species richness, species' distribution patterns and availability of historical information. This work aims at providing a comprehensive picture of the distribution and abundance of intertidal macro-invertebrates and macro-algae along the Portuguese rocky coast in the early 2000s.

**New information:**

This study provides a description of the rocky shore intertidal biodiversity of the mainland Portuguese coast in the early 2000s. The spatial distribution and semi-quantitative abundance of a total of 238 taxa were assessed at 49 wave-exposed locations. These data provide a comprehensive baseline against which biodiversity changes can be effectively and objectively evaluated.

## Introduction

Biodiversity is now declining globally at rates unprecedented in human history (Ceballos et al. 2017, IPBES 2019). This process is being mainly driven by habitat degradation and loss and compounded by the recent climate change (IPCC 2014). Biodiversity loss has been severely affecting species distributions, community structure, ecosystem function, ecosystem services, food security and public health (Edwards and Richardson 2004, Hawkins et al. 2008, Wernberg et al. 2011, IPBES 2019). This so-called "biodiversity crisis" has motivated a renewed interest in effective environmental and biodiversity monitoring (Navarro et al. 2017, CBD 2010). Broad-scale, sustained observations, including historical data rescue, are now regarded as essential to understand past trends and to produce accurate forecasts which are needed to provide information for policy decisions (Hawkins et al. 2013).

Intertidal systems are well suited for studies focusing on the effects of climate variability and climate change on biodiversity, as they are amongst the most thermally complex environments on Earth (Pincebourde et al. 2008) and are strongly influenced by meteorological conditions (Bates et al. 2018, Zamir et al. 2018). They are inhabited by marine organisms that must withstand terrestrial conditions during low tide. Stressful events may have dire consequences for these species (Jurgens et al. 2015), which are, therefore, regarded as sensitive indicators of climate variability and change (Helmuth et al. 2006).

Stemming from its peculiar geographic, climatic and oceanographic setting, the biogeography of the Portuguese rocky coast is quite interesting and offers exceptional conditions for studying the effects of climate change on the distribution of species. First, the coast is mostly linear with a north-to-south orientation and with a latitudinal gradient in temperature during the winter, from relatively cold water in the north to relatively warm water in the south. In the summer, that latitudinal gradient is often intensified by the effects of coastal upwelling, which brings deep cold water to the surface near the coast, especially in the northern portion of the country (Lemos and Pires 2004, Lima et al. 2007, Seabra et al. 2015). These clinal variations in temperature result in a region of contact between meridional (warm-water) and septentrional (cold-water) fauna and flora, where the distribution limits of several species are reached (Fischer-Piétte 1959, Fischer-Piétte and Gaillard 1959, Fischer-Piétte 1963, Ardré 1970, Santos 2000, Araújo et al. 2009, Lima et al. 2007). Second and also due to the cold effects of upwelling, the northern Portuguese coast is, on the one hand, a biogeographic enclave for cold-water species, such as *Himanthaliaelongata*, *Saccharinalatissima* or *Pelvetiacanaliculata*, which can only be found again thousands of kilometres further north, in Brittany (France). On the other hand, many warm-water species, such as *Codiumadhaerens*, *Padina pavonica* or *Valoniautricularis*, occur both towards either the north or the south, but not in this region; in other words, their distribution features a prominent gap in northern Portugal (van den Hoek and and Donze 1967, Lima et al. 2007). Third, shifts in species distributions have been described since the 1950s, not only for this particular stretch of coastline, but also for the neighbouring regions in the Iberian Peninsula (Fischer-Piétte and Forest 1951, Fischer-Piétte 1957, [Bibr B7184946][Bibr B7184982], Ardré 1971, Santos 2000, Lima et al. 2006, Lima et al. 2007, Berke et al. 2010, Wethey et al. 2011, Rubal et al. 2013). Fourth, recent evidence suggests that, due to the effects of coastal upwelling, the Portuguese coast has been warming at a slower pace than its neighbouring regions, but that effect may become compromised in the near future (Varela et al. 2018, Seabra et al. 2019). Thus, the long-term monitoring of this region is essential to understand if changes in the distribution of its inhabiting species are either the result of climate change or local impacts (Hawkins 2012).

This work aims at providing a comprehensive picture of the distribution and abundance of intertidal macro-invertebrates and macro-algae along the Portuguese rocky coast in the early 2000s. These data, previously unpublished, may be used for environmental management (e.g. as an aid for the decision processes leading to the establishment of coastal protection areas), in conservation contexts (e.g. for environmental impact assessments studies), in ecological studies (e.g. to better understand the complex relationships between environment and biodiversity) or in climate change studies (since alterations in species distributions may be used as warning signs of the effects of climate change).

## Sampling methods

### Study extent

**Figure 1.**
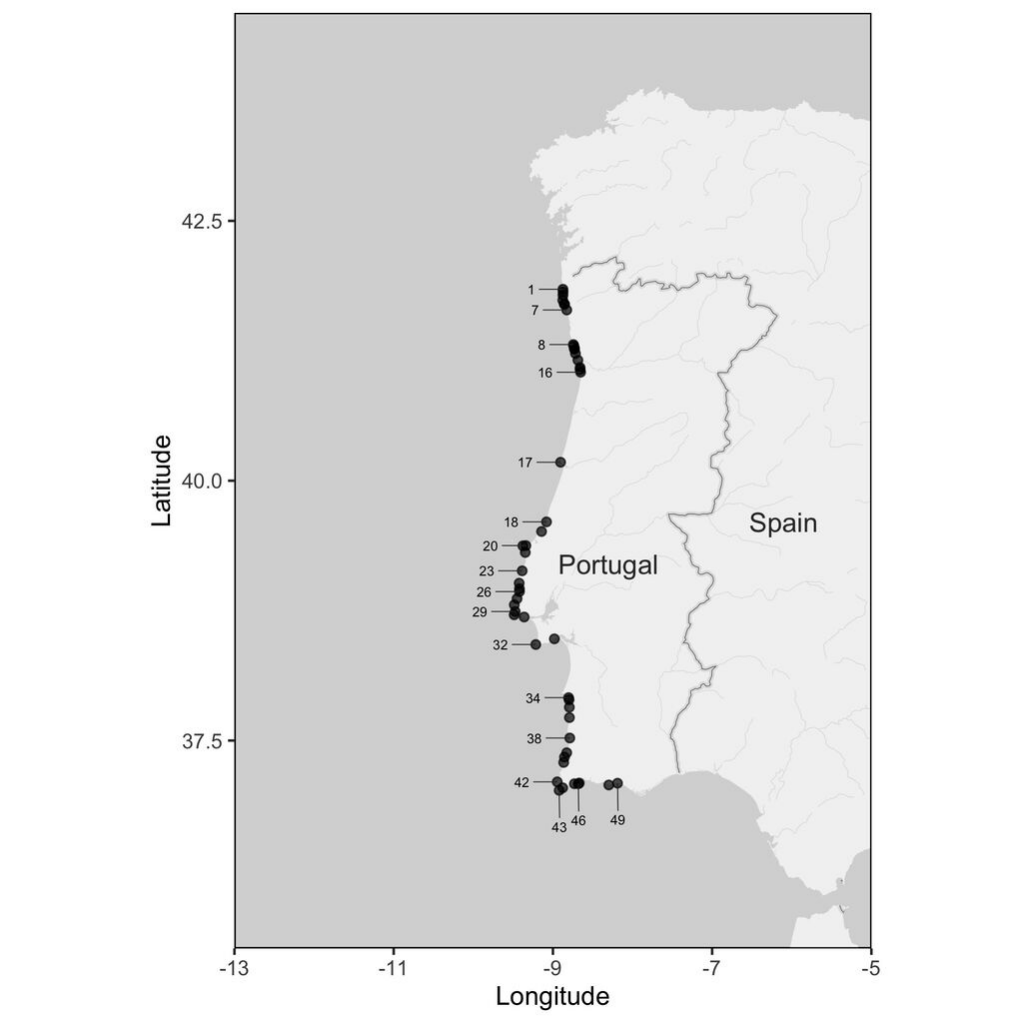
Study locations along the Portuguese coast (Western Iberia) visited in the years of 2001 and 2002. Location details and sampling dates can be found in Table 1.

**Figure 2.**
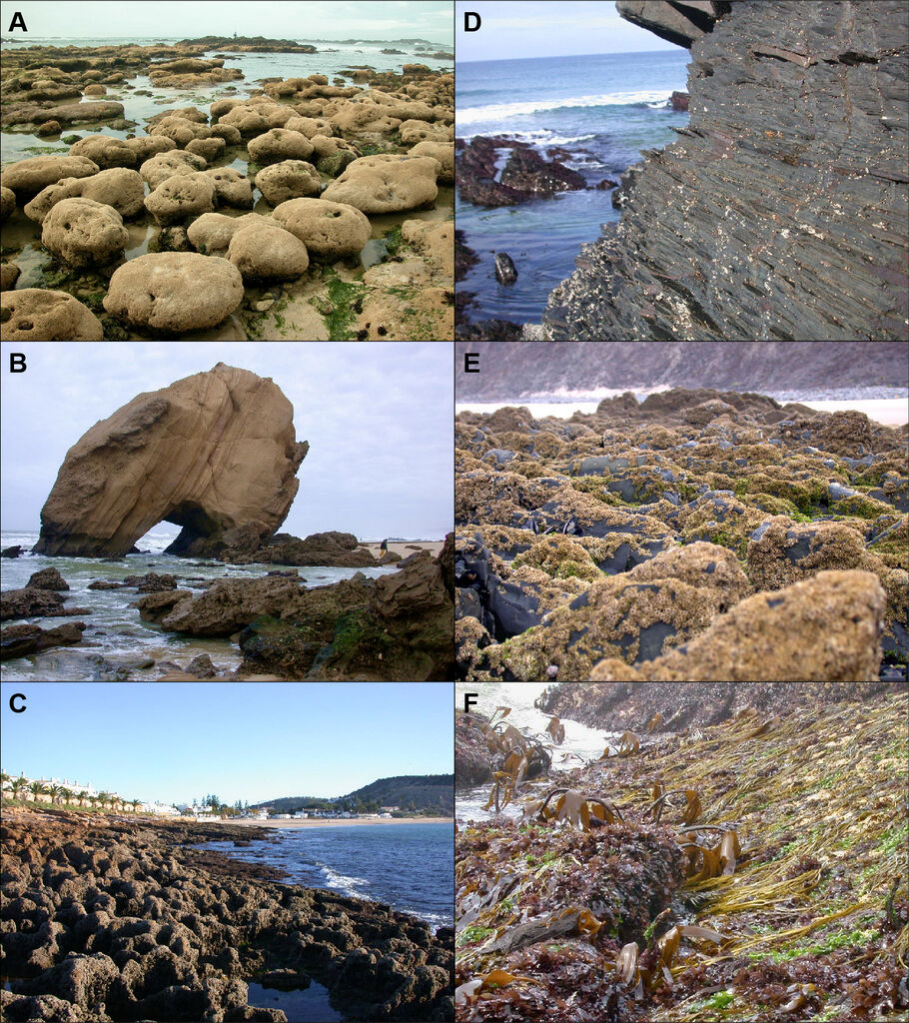
Examples of shores and tide levels surveyed in the present study. A - Mindelo, in northern Portugal, B - Santa Cruz, in central Portugal and, C - Dona Ana, in southern Portugal. D - High intertidal at Monte Clérigo, with *Melarhapheneritoides*, E - Mid-intertidal at Arrifana, with barnacles and mussels, F - Low intertidal at Mindelo featuring *Himanthaliaelongata*, which is now almost extinct from the area. Photos taken by Fernando P. Lima.

Data were collected during the autumns of 2001 and 2002 (Table 1). Sampling was carried out at 49 wave-exposed rocky shore locations along the Portuguese coast, covering the three major rocky stretches of shoreline (Fig. 1, Fig. 2, Table 1). In the northern region, we sampled the locations of Moledo do Minho, Vila Praia de Âncora, Afife, Montedor, Forte da Vigia, Praia Norte, Amorosa, Mindelo, Vila Chã, Labruje, Angeiras, Cabo do Mundo, Homem do Leme, Valadares, Miramar and Aguda. In the central region, we sampled Nazaré, São Martinho do Porto, Baleal, Papoa, São Bernardino, Santa Cruz, São Lourenço, Ericeira, São Julião, Magoito, Adraga, Abano, Cabo Raso, Avencas, Cabo Espichel and Portinho da Arrábida. In the southern region, we sampled São Torpes, Oliveirinha, Queimado, Vila Nova de Milfontes, Zambujeira do Mar, Vale dos Homens, Monte Clérigo, Arrifana, Castelejo, Martinhal, Ingrina, Praia da Luz, Porto de Mós, Dona Ana, Castelo and Olhos de Água. The location of Buarcos, roughly at the mid-point between the northern and the central stretches of rocky coast, was also included in this study.

### Sampling description

**Figure 3.**
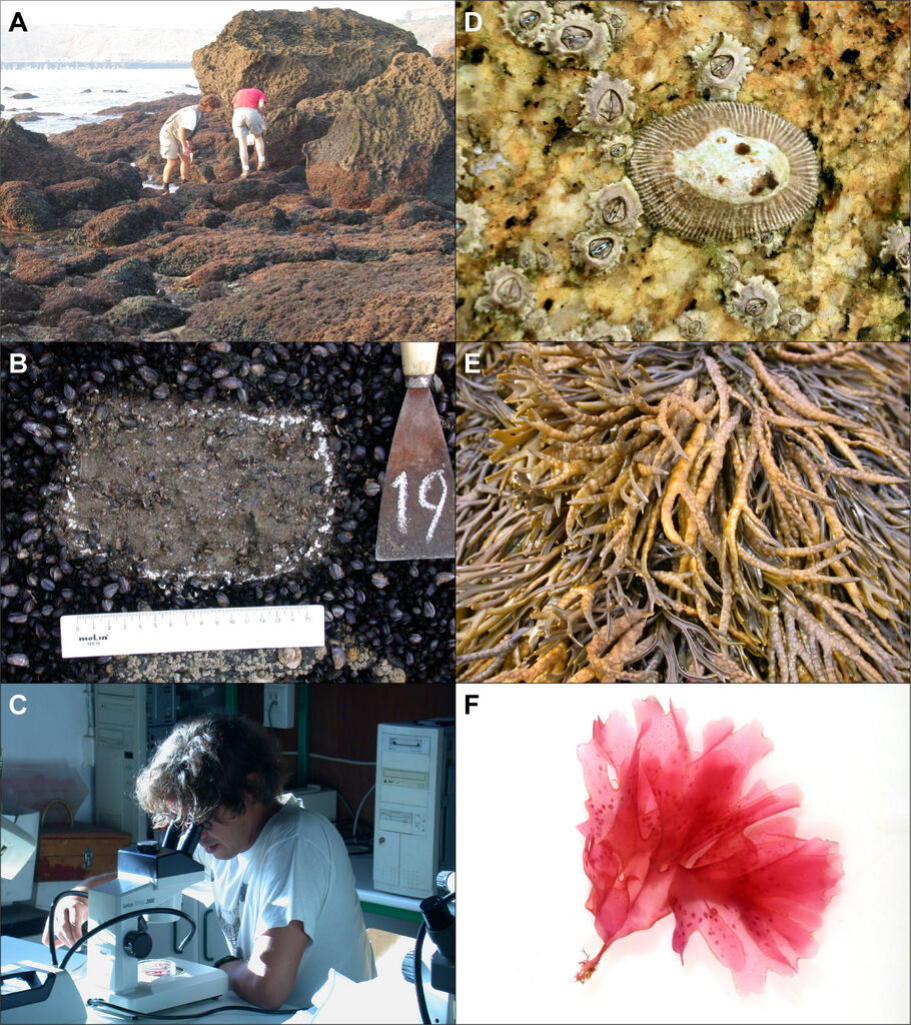
A - A team of two people performing the SACFOR survey at Martinhal, B - Substrate scraping in a mussel bed, C - Identification of species in the lab under a stereomicroscope, D - *Siphonariapectinata*, an invasive warm-water species amongst *Chthamalusmontagui* in a tide pool, E - *Pelvetiacanaliculata*, a cold-water species with its southern distribution limit at Cabo do Mundo, F - The red algae *Nitophyllumpunctatum* as seen under a stereomicroscope. Photos taken by Fernando P. Lima.

Each of the 49 studied locations were extensively surveyed from the splash fringe level in the high intertidal (area of occurrence of littorinids and lichens) to the low fringe level at the low intertidal (area of occurrence of red, green and brown algae, see Fig. 2). At each location, a two-people team worked from one hour before the low tide peak to one hour after low tide (Fig. 3). The average astronomical low tide height during surveys was -1.51 ± 0.14 m below mean sea water level (Table 1). The occurrence and abundance of all easily-identified taxa (animals and algae, sensu latu) were recorded in situ. A semi-quantitative estimation of abundance was assigned to each taxa identified during the survey. We used a modified version of the scale established by Crisp and Southward (1958) — **SACFOR**, where abundances were encoded from 6 to 0 (where 6 means **S**uperabundant; 5, **A**bundant; 4, **C**ommon; 3, **F**requent; 2, **O**ccasional; 1, **R**are; and 0, not found). Small animals or algae (turfs) or other taxa of dubious classification were collected and their identification finalised in the lab under a stereomicroscope (Fig. 3). Additionally, whole substrate samples were collected by scraping the substrate with a paint scraper spatula at three tidal levels: (1) amongst barnacles, (2) amongst mussels and honeycomb worm reefs (*Sabellariaalveolata*) and (3) amongst red, green and brown algae and preserved in a solution of 4% formaldehyde in seawater (Fig. 3). The scraped area was approximately 150-225 cm^2^ per sample. The number of samples varied between four and six, accordingly to the spatial heterogeneity of each substrate, but totalling approximately 900 cm^2^ per shore and substrate. Later, in the lab, formaldehyde was removed from samples by washing them with running water and smaller organisms were separated from larger mussels, honeycomb worm reefs or canopy algae using a 0.25 cm mesh sieve and identified to the lowest taxonomic level possible.

### Quality control

In addition to AlgaeBase (Guiry and Guiry 2021), authoritative identification guides and keys for the Eastern Atlantic and Mediterranean were used. Specifically, Dixon and Irvine (1977), Hiscock (1979), Irvine (1983), Bárbara and Cremades (1987), Christensen (1987), Fletcher (1987), Saldanha (1988), Burrows (1991), Cabioc'h et al. (1992), Maggs and Hommersand (1993), Hiscock (1986), Irvine and Chamberlain (1994), Brodie and Irvine (2003) were used for algae and Naylor (1972), Lincoln (1979), Manuel (1981), Graham (1988), Hayward and Ryland (1991) were used for animals. All scientific names were standardised against the WoRMS - The World Register of Marine Species using the Taxon Match tool available at http://www.marinespecies.org/aphia.php?p=match (accessed on: 07-07-2021).

### Step description

The steps that led to the final release of the dataset were as follows: (1) In-situ identification of species and attribution of a semi-quantitative abundance SACFOR score; (2) destructive sampling (substrate scraping) at three tidal levels and preservation of samples in formaldehyde; (3) cleaning of formaldehyde, sorting and identification of specimens in the lab; (4) conversion of paper-based records from the field and from the lab into an electronic data format (speadsheets); (5) integration of the field and laboratory datasets into a standardised format; (6) retrieval of missing geographical information, georeferencing of coordinates through Google Earth and general quality control; (7) standardisation of taxonomy against the World Register of Marine Species; (8) export of data as a DarwinCore Archive and (9) generation of dataset-level metadata.

## Geographic coverage

### Description

Sampling was done along the three major rocky stretches of the entire coast of mainland Portugal, covering an extension of approximately 700 km from Moledo do Minho to Olhos de Água.

### Coordinates

37.021090 and 41.841824 Latitude; -9.486673 and -8.188497 Longitude.

## Taxonomic coverage

### Taxa included

**Table:** 

Rank	Scientific Name	
kingdom	Plantae	
kingdom	Animalia	
kingdom	Chromista	
kingdom	Bacteria	
kingdom	Fungi	

## Temporal coverage

**Data range:** 2001-9-16 – 2002-12-06.

## Usage licence

### Usage licence

Open Data Commons Attribution License

### IP rights notes

Data users are free to share, create and adapt the dataset as long as they adequately attribute (cite) this work.

## Data resources

### Data package title

Intertidal Biodiversity along the Portuguese Coast (2001-2002)

### Resource link

http://ipt.gbif.pt/ipt/resource?r=ibpc

### Alternative identifiers

https://doi.org/10.15468/mbg5p3

### Number of data sets

1

### Data set 1.

#### Data set name

Intertidal Biodiversity along the Portuguese coast (2001-2002)

#### Data format

Darwin Core archive

#### Number of columns

38

#### Download URL

http://ipt.gbif.pt/ipt/resource?r=ibpc

#### Description

The data presented in this paper derives from visual and destructive surveys done along the Portuguese coast in the early 2000s. The dataset published in GBIF has the structure of a Sampling event dataset with two data subsets: Events (Core) and Associated occurrences. These data have been published as a Darwin Core Archive (DwCA), which is a standardised format for sharing biodiversity data. The Sampling Event (Core) contains 49 records (eventID). The extension data (Associated Occurrences) sheet has 11662 occurrences.

**Data set 1.** 

Column label	Column description
eventID	Unique identifier associated with an event
samplingProtocol	Sampling method used during the event
samplingEffort	Description of effort during the sampling event
eventDate	The date of the event
year	The year of the event
month	The month of the event
day	The day of the event
eventRemarks	Astronomical low tide height during the event
country	Country where the event took place
countryCode	The unique code of the country where the event took place
locationID	An identifier for the location information from Geonames
decimalLatitude	The geographical latitude of the event
decimalLongitude	The geographical longitude of the event
geodeticDatum	The geodetic datum upon which the geographical coordinates are based
coordinatePrecision	The precision of the coordinates
coordinateUncertaintyInMetres	The uncertainty of the coordinates, in metres
type	Type of dataset
ownerInstitutionCode	Identifier code of the owner institution
habitat	The habitat in which the event took place
waterBody	The water body in which the event took place
rightsHolder	The rights holder of the dataset
bibliographicCitation	Bibliographic citation of the dataset publication
occurrenceID	Unique identifier associated with the occurrence of a species
basisOfRecord	The specific nature of the data record
organismQuantity	An enumeration value for the quantity of a species
organismQuantityType	The quantification scale of the quantity of a species
occurrenceStatus	A statement about the presence or absence of a species in a location
scientificName	The full scientific name, with authorship and date information, if known
scientificNameID	Unique identifier of a species, obtained from WoRMS
kingdom	The full scientific name of the kingdom in which the taxon is classified
phylum	The full scientific name of the phylum in which the taxon is classified
class	The full scientific name of the class in which the taxon is classified
order	The full scientific name of the order in which the taxon is classified
family	The full scientific name of the family in which the taxon is classified
genus	The full scientific name of the genus in which the taxon is classified
specificEpithet	The specific epithet of the species
taxonRank	The taxonomic rank of the most specific name in scientificName
recordedBy	Person(s) responsible for sampling the occurrence

## Additional information

A total of 238 taxa (Table 2) were identified: 99 Plantae, 36 Chromista, 100 Animalia, two Fungi and one Bacterium (Pereira et al. 2021). A description of number of taxa of each Phylum per location is present in Table 3. The site with the least amount of species was Adraga and the one with the most was Nazaré, both sites in central Portugal. On average, the surveyed locations in northern Portugal had a higher number of species (67 species per location), followed by the locations in southern Portugal (66 species per site). On average, locations in central Portugal (59 species per site) had the lowest number of species (Table 3).

## Figures and Tables

**Table 1.** Names of the locations visited in this study, sampling date, coordinates and low tide height on the day of sampling. Map reference numbers are cross-referenced with Fig. 1.

**Map reference**	**Location**	**Sampling date**	**Latitude**	**Longitude**	**Astronomical low tide height (m below mean sea water level)**
1	Moledo do Minho	2001-09-19	41.841824	-8.873979	-1.70
2	Vila Praia de Âncora	2001-09-20	41.816049	-8.872269	-1.60
3	Afife	2002-11-07	41.784753	-8.873573	-1.58
4	Montedor	2001-09-20	41.736517	-8.876095	-1.60
5	Forte da Vigia	2001-09-19	41.699426	-8.857093	-1.71
6	Praia Norte	2002-11-06	41.698016	-8.853935	-1.69
7	Amorosa	2002-06-25	41.642586	-8.825918	-1.24
8	Mindelo	2001-09-17	41.309900	-8.742765	-1.57
9	Vila Chã	2001-09-17	41.295714	-8.737265	-1.57
10	Labruje	2002-11-05	41.274929	-8.729791	-1.69
11	Angeiras	2002-11-05	41.264639	-8.729113	-1.69
12	Cabo do Mundo	2001-09-18	41.223489	-8.716956	-1.68
13	Homem do Leme	2001-09-18	41.159700	-8.686061	-1.68
14	Valadares	2001-09-16	41.088863	-8.658663	-1.40
15	Miramar	2002-11-04	41.068286	-8.658869	-1.58
16	Aguda	2001-09-16	41.043782	-8.652202	-1.40
17	Buarcos	2002-01-02	40.177513	-8.903539	-1.44
18	Nazaré	2001-10-19	39.603837	-9.080406	-1.48
19	São Martinho do Porto	2001-10-19	39.511391	-9.142621	-1.48
20	Baleal	2001-10-18	39.375856	-9.339807	-1.59
21	Papoa	2001-10-18	39.373440	-9.377283	-1.58
22	São Bernardino	2002-12-03	39.309556	-9.347106	-1.42
23	Santa Cruz	2002-12-04	39.132919	-9.386206	-1.51
24	São Lourenço	2002-12-04	39.014081	-9.423544	-1.50
25	Ericeira	2001-10-17	38.961181	-9.420290	-1.59
26	São Julião	2002-12-04	38.933212	-9.420080	-1.49
27	Magoito	2001-10-17	38.865024	-9.450610	-1.58
28	Adraga	2002-12-06	38.805798	-9.485069	-1.40
29	Abano	2002-12-05	38.741236	-9.473426	-1.48
30	Cabo raso	2001-10-16	38.709571	-9.486673	-1.47
31	Avencas	2001-10-16	38.688432	-9.360986	-1.49
32	Cabo Espichel	2001-12-06	38.423685	-9.215187	-0.91
33	Portinho da Arrábida	2002-12-06	38.478248	-8.981859	-1.39
34	São Torpes	2002-10-08	37.912645	-8.802884	-1.63
35	Oliveirinha	2001-11-17	37.891804	-8.797249	-1.35
36	Queimado	2002-10-08	37.820059	-8.793436	-1.63
37	Vila Nova de Milfontes	2001-11-17	37.720834	-8.792484	-1.35
38	Zambujeira do Mar	2001-11-16	37.524209	-8.788036	-1.45
39	Vale dos Homens	2002-10-07	37.382567	-8.826646	-1.64
40	Monte Clérigo	2001-11-16	37.340579	-8.854811	-1.44
41	Arrifana	2002-10-07	37.289191	-8.864691	-1.64
42	Castelejo	2001-11-15	37.102461	-8.945659	-1.45
43	Martinhal	2002-10-06	37.021090	-8.921792	-1.53
44	Ingrina	2001-11-15	37.045155	-8.877728	-1.45
45	Praia da Luz	2001-11-14	37.084187	-8.729989	-1.38
46	Porto de Mós	2002-10-06	37.083850	-8.681465	-1.53
47	Dona Ana	2001-11-14	37.091115	-8.668970	-1.38
48	Castelo	2002-10-05	37.072434	-8.298182	-1.38
49	Olhos de Água	2002-10-05	37.089327	-8.188497	-1.38

**Table 2.** List of species surveyed, scientifc name ID from the World Register of Marine Species (WoRMS) and taxonomic ranks.

**Scientific name**	**Scientific name ID (Worms)**	**Order**	**Family**
*Eulaliaviridis* (Linnaeus, 1767)	urn:lsid:marinespecies.org:taxname:130639	Phyllodocida	Phyllodocidae
*Filogranaimplexa* (Berkeley, 1835)	urn:lsid:marinespecies.org:taxname:130989	Sabellida	Serpulidae
*Sabellaspallanzanii* (Gmelin, 1791)	urn:lsid:marinespecies.org:taxname:130969	Sabellida	Sabellidae
*Sabellariaalveolata* (Linnaeus, 1767)	urn:lsid:marinespecies.org:taxname:130866		Sabellariidae
*Spirobranchustriqueter* (Linnaeus, 1758)	urn:lsid:marinespecies.org:taxname:131027	Sabellida	Serpulidae
*Austrominiusmodestus* (Darwin, 1854)	urn:lsid:marinespecies.org:taxname:106209	Balanomorpha	Elminiidae
*Cancerpagurus* (Linnaeus, 1758)	urn:lsid:marinespecies.org:taxname:107276	Decapoda	Cancridae
*Carcinusmaenas* (Linnaeus, 1758)	urn:lsid:marinespecies.org:taxname:107381	Decapoda	Carcinidae
*Chthamalusmontagui* (Southward, 1976)	urn:lsid:marinespecies.org:taxname:106230	Balanomorpha	Chthamalidae
*Chthamalusstellatus* (Poli, 1791)	urn:lsid:marinespecies.org:taxname:106231	Balanomorpha	Chthamalidae
*Diogenespugilator* (P. Roux, 1829)	urn:lsid:marinespecies.org:taxname:107199	Decapoda	Diogenidae
*Eriphiaverrucosa* (Forskål, 1775)	urn:lsid:marinespecies.org:taxname:107409	Decapoda	Eriphiidae
*Galatheastrigosa* (Linnaeus, 1761)	urn:lsid:marinespecies.org:taxname:107155	Decapoda	Galatheidae
*Ligiaitalica* (Fabricius, 1798)	urn:lsid:marinespecies.org:taxname:156211	Isopoda	Ligiidae
*Ligiaoceanica* (Linnaeus, 1767)	urn:lsid:marinespecies.org:taxname:146999	Isopoda	Ligiidae
*Lophozozymusincisus* (H. Milne Edwards, 1834)	urn:lsid:marinespecies.org:taxname:444382	Decapoda	Xanthidae
*Majasquinado* (Herbst, 1788)	urn:lsid:marinespecies.org:taxname:107350	Decapoda	Majidae
*Necorapuber* (Linnaeus, 1767)	urn:lsid:marinespecies.org:taxname:107398	Decapoda	Polybiidae
*Pachygrapsusmarmoratus* (J.C. Fabricius, 1787)	urn:lsid:marinespecies.org:taxname:107455	Decapoda	Grapsidae
*Palaemonserratus* (Pennant, 1777)	urn:lsid:marinespecies.org:taxname:107616	Decapoda	Palaemonidae
*Perforatusperforatus* (Bruguière, 1789)	urn:lsid:marinespecies.org:taxname:106219	Balanomorpha	Balanidae
*Pilumnushirtellus* (Linnaeus, 1761)	urn:lsid:marinespecies.org:taxname:107418	Decapoda	Pilumnidae
*Pirimeladenticulata* (Montagu, 1808)	urn:lsid:marinespecies.org:taxname:107278	Decapoda	Carcinidae
*Pollicipespollicipes* (Gmelin, 1791)	urn:lsid:marinespecies.org:taxname:106177	Pollicipedomorpha	Pollicipedidae
*Porcellanaplatycheles* (Pennant, 1777)	urn:lsid:marinespecies.org:taxname:107190	Decapoda	Porcellanidae
*Lichinapygmaea* (Lightf.) C. Agardh, 1817	urn:lsid:marinespecies.org:taxname:147720	Lichinales	Lichinaceae
*Verrucariamaura* (Wahlenberg, 1803)	urn:lsid:marinespecies.org:taxname:147758	Verrucariales	Verrucariaceae
*Electrapilosa* (Linnaeus, 1767)	urn:lsid:marinespecies.org:taxname:111355	Cheilostomatida	Electridae
*Turbicelleporaavicularis* (Hincks, 1860)	urn:lsid:marinespecies.org:taxname:111285	Cheilostomatida	Celleporidae
*Bryopsis* (J.V.Lamouroux, 1809)	urn:lsid:marinespecies.org:taxname:143812	Bryopsidales	Bryopsidaceae
*Cladophora* (Kützing, 1843)	urn:lsid:marinespecies.org:taxname:143996	Cladophorales	Cladophoraceae
*Codiumadhaerens* (C.Agardh, 1822)	urn:lsid:marinespecies.org:taxname:145078	Bryopsidales	Codiaceae
*Codiumbursa* (Olivi) C.Agardh, 1817	urn:lsid:marinespecies.org:taxname:145079	Bryopsidales	Codiaceae
*Codiumtomentosum* (Stackhouse, 1797)	urn:lsid:marinespecies.org:taxname:145092	Bryopsidales	Codiaceae
*Ulva* (Linnaeus, 1753)	urn:lsid:marinespecies.org:taxname:144296	Ulvales	Ulvaceae
*Valonia* (C.Agardh, 1823)	urn:lsid:marinespecies.org:taxname:144267	Cladophorales	Valoniaceae
*Coryphoblenniusgalerita* (Linnaeus, 1758)	urn:lsid:marinespecies.org:taxname:126762	Blenniiformes	Blenniidae
*Diplodus* (Rafinesque, 1810)	urn:lsid:marinespecies.org:taxname:126076	Eupercaria incertae sedis	Sparidae
*Gaidropsarusmediterraneus* (Linnaeus, 1758)	urn:lsid:marinespecies.org:taxname:126457	Gadiformes	Lotidae
*Gobiuspaganellus* (Linnaeus, 1758)	urn:lsid:marinespecies.org:taxname:126893	Gobiiformes	Gobiidae
*Lepadogaster* (Goüan, 1770)	urn:lsid:marinespecies.org:taxname:125781	Gobiesociformes	Gobiesocidae
*Lipophryspholis* (Linnaeus, 1758)	urn:lsid:marinespecies.org:taxname:126768	Blenniiformes	Blenniidae
*Nerophislumbriciformis* (Jenyns, 1835)	urn:lsid:marinespecies.org:taxname:127383	Syngnathiformes	Syngnathidae
*Parablenniusgattorugine* (Linnaeus, 1758)	urn:lsid:marinespecies.org:taxname:126770	Blenniiformes	Blenniidae
*Salariapavo* (Risso, 1810)	urn:lsid:marinespecies.org:taxname:302108	Blenniiformes	Blenniidae
*Actiniaequina* (Linnaeus, 1758)	urn:lsid:marinespecies.org:taxname:100803	Actiniaria	Actiniidae
*Actiniafragacea* Tugwell, 1856	urn:lsid:marinespecies.org:taxname:100805	Actiniaria	Actiniidae
*Actinothoesphyrodeta* (Gosse, 1858)	urn:lsid:marinespecies.org:taxname:100986	Actiniaria	Sagartiidae
*Anemoniaviridis* (Forsskål, 1775)	urn:lsid:marinespecies.org:taxname:100808	Actiniaria	Actiniidae
*Anthopleurathallia* (Gosse, 1854)	urn:lsid:marinespecies.org:taxname:100812	Actiniaria	Actiniidae
*Aulactiniaverrucosa* (Pennant, 1777)	urn:lsid:marinespecies.org:taxname:100819	Actiniaria	Actiniidae
Caryophyllia (Caryophyllia) smithii (Stokes & Broderip, 1828)	urn:lsid:marinespecies.org:taxname:1288958	Scleractinia	Caryophylliidae
*Cereuspedunculatus* (Pennant, 1777)	urn:lsid:marinespecies.org:taxname:100987	Actiniaria	Sagartiidae
*Clytiahemisphaerica* (Linnaeus, 1767)	urn:lsid:marinespecies.org:taxname:152074	Leptothecata	Campanulariidae
Corynactisviridis (Allman, 1846)	urn:lsid:marinespecies.org:taxname:101016	Corallimorpharia	Corallimorphidae
*Obeliageniculata* (Linnaeus, 1758)	urn:lsid:marinespecies.org:taxname:117388	Leptothecata	Campanulariidae
*Urticinafelina* (Linnaeus, 1761)	urn:lsid:marinespecies.org:taxname:100798	Actiniaria	Actiniidae
Calothrix C.Agardh ex Bornet & Flahault, 1886	urn:lsid:marinespecies.org:taxname:146624	Nostocales	Rivulariaceae
*Asteriasrubens* (Linnaeus, 1758)	urn:lsid:marinespecies.org:taxname:123776	Forcipulatida	Asteriidae
*Asterinagibbosa* (Pennant, 1777)	urn:lsid:marinespecies.org:taxname:123987	Valvatida	Asterinidae
*Coscinasteriastenuispina* (Lamarck, 1816)	urn:lsid:marinespecies.org:taxname:123795	Forcipulatida	Asteriidae
*Holothuria* (Linnaeus, 1767)	urn:lsid:marinespecies.org:taxname:123456	Holothuriida	Holothuriidae
Holothuria (Panningothuria) forskali Delle (Chiaje, 1823)	urn:lsid:marinespecies.org:taxname:124501	Holothuriida	Holothuriidae
*Marthasteriasglacialis* (Linnaeus, 1758)	urn:lsid:marinespecies.org:taxname:123803	Forcipulatida	Asteriidae
*Ophiocominanigra* (Abildgaard in O.F. Müller, 1789)	urn:lsid:marinespecies.org:taxname:125027	Ophiacanthida	Ophiotomidae
*Ophiothrixfragilis* (Abildgaard in O.F. Müller, 1789)	urn:lsid:marinespecies.org:taxname:125131	Amphilepidida	Ophiotrichidae
*Paracentrotuslividus* (Lamarck, 1816)	urn:lsid:marinespecies.org:taxname:124316	Camarodonta	Parechinidae
*Acanthochitonacrinita* (Pennant, 1777)	urn:lsid:marinespecies.org:taxname:138675	Chitonida	Acanthochitonidae
*Aplysia* (Linnaeus, 1767)	urn:lsid:marinespecies.org:taxname:137654	Aplysiida	Aplysiidae
*Aplysiafasciata* (Poiret, 1789)	urn:lsid:marinespecies.org:taxname:138755	Aplysiida	Aplysiidae
*Bolmarugosa* (Linnaeus, 1767)	urn:lsid:marinespecies.org:taxname:751225	Trochida	Turbinidae
*Calliostomazizyphinum* (Linnaeus, 1758)	urn:lsid:marinespecies.org:taxname:141767	Trochida	Calliostomatidae
*Conus* (Linnaeus, 1758)	urn:lsid:marinespecies.org:taxname:137813	Neogastropoda	Conidae
*Diodoragraeca* (Linnaeus, 1758)	urn:lsid:marinespecies.org:taxname:139951	Lepetellida	Fissurellidae
*Dorispseudoargus* (Rapp, 1827)	urn:lsid:marinespecies.org:taxname:138763	Nudibranchia	Dorididae
*Gibbulamagus* (Linnaeus, 1758)	urn:lsid:marinespecies.org:taxname:141790	Trochida	Trochidae
*Hiatellaarctica* (Linnaeus, 1767)	urn:lsid:marinespecies.org:taxname:140103	Adapedonta	Hiatellidae
*Lasaearubra* (Montagu, 1803)	urn:lsid:marinespecies.org:taxname:140176	Galeommatida	Lasaeidae
*Littorinalittorea* (Linnaeus, 1758)	urn:lsid:marinespecies.org:taxname:140262	Littorinimorpha	Littorinidae
*Littorinaobtusata* (Linnaeus, 1758)	urn:lsid:marinespecies.org:taxname:140263	Littorinimorpha	Littorinidae
*Littorinasaxatilis* (Olivi, 1792)	urn:lsid:marinespecies.org:taxname:140264	Littorinimorpha	Littorinidae
*Melarhapheneritoides* (Linnaeus, 1758)	urn:lsid:marinespecies.org:taxname:151586	Littorinimorpha	Littorinidae
*Modiolusbarbatus* (Linnaeus, 1758)	urn:lsid:marinespecies.org:taxname:140464	Mytilida	Mytilidae
*Musculuscostulatus* (Risso, 1826)	urn:lsid:marinespecies.org:taxname:140471	Mytilida	Mytilidae
*Mytilusgalloprovincialis* (Lamarck, 1819	urn:lsid:marinespecies.org:taxname:140481	Mytilida	Mytilidae
*Nucellalapillus* (Linnaeus, 1758)	urn:lsid:marinespecies.org:taxname:140403	Neogastropoda	Muricidae
*Ocenebraerinaceus* (Linnaeus, 1758)	urn:lsid:marinespecies.org:taxname:140405	Neogastropoda	Muricidae
*Octopusvulgaris* (Cuvier, 1797)	urn:lsid:marinespecies.org:taxname:140605	Octopoda	Octopodidae
*Onchidellaceltica* (Audouin & Milne-Edwards, 1832)	urn:lsid:marinespecies.org:taxname:140626	Systellommatophora	Onchidiidae
*Ostreaedulis* (Linnaeus, 1758)	urn:lsid:marinespecies.org:taxname:140658	Ostreida	Ostreidae
*Patellaaspera* (Röding, 1798)	urn:lsid:marinespecies.org:taxname:456570		Patellidae
*Patelladepressa* (Pennant, 1777)	urn:lsid:marinespecies.org:taxname:151374		Patellidae
*Patellapellucida* (Linnaeus, 1758)	urn:lsid:marinespecies.org:taxname:162669		Patellidae
*Patellarustica* (Linnaeus, 1758)	urn:lsid:marinespecies.org:taxname:140683		Patellidae
*Patellavulgata* (Linnaeus, 1758)	urn:lsid:marinespecies.org:taxname:140685		Patellidae
*Phorcuslineatus* (da Costa, 1778)	urn:lsid:marinespecies.org:taxname:153534	Trochida	Trochidae
*Sepiaofficinalis* (Linnaeus, 1758)	urn:lsid:marinespecies.org:taxname:141444	Sepiida	Sepiidae
*Siphonariapectinata* (Linnaeus, 1758)	urn:lsid:marinespecies.org:taxname:141470	Siphonariida	Siphonariidae
*Steromphalacineraria* (Linnaeus, 1758)	urn:lsid:marinespecies.org:taxname:141782	Trochida	Trochidae
*Steromphalapennanti* (Philippi, 1846)	urn:lsid:marinespecies.org:taxname:141792	Trochida	Trochidae
*Steromphalaumbilicalis* (da Costa, 1778)	urn:lsid:marinespecies.org:taxname:141801	Trochida	Trochidae
*Stramonitahaemastoma* (Linnaeus, 1767)	urn:lsid:marinespecies.org:taxname:224350	Neogastropoda	Muricidae
*Trapaniamaculata* (Haefelfinger, 1960)	urn:lsid:marinespecies.org:taxname:140044	Nudibranchia	Goniodorididae
*Tricoliapullus* (Linnaeus, 1758)	urn:lsid:marinespecies.org:taxname:141700	Trochida	Phasianellidae
*Tritiaincrassata* (Strøm, 1768)	urn:lsid:marinespecies.org:taxname:140503	Neogastropoda	Nassariidae
*Tritiareticulata* (Linnaeus, 1758)	urn:lsid:marinespecies.org:taxname:140513	Neogastropoda	Nassariidae
*Triviamonacha* (da Costa, 1778)	urn:lsid:marinespecies.org:taxname:141744	Littorinimorpha	Triviidae
*Alariaesculenta* (Linnaeus) Greville, 1830	urn:lsid:marinespecies.org:taxname:145716	Laminariales	Alariaceae
*Ascophyllumnodosum* (Linnaeus) Le Jolis, 1863	urn:lsid:marinespecies.org:taxname:145541	Fucales	Fucaceae
*Bifurcariabifurcata* (R.Ross, 1958)	urn:lsid:marinespecies.org:taxname:145503	Fucales	Sargassaceae
*Cladostephusspongiosus* (Hudson) C.Agardh, 1817	urn:lsid:marinespecies.org:taxname:145888	Sphacelariales	Cladostephaceae
*Colpomeniaperegrina* (Sauvageau, 1927)	urn:lsid:marinespecies.org:taxname:145856	Ectocarpales	Scytosiphonaceae
*Cystoseira* (C.Agardh, 1820)	urn:lsid:marinespecies.org:taxname:144126	Fucales	Sargassaceae
*Cystoseirahumilis* (Schousboe ex Kützing, 1860)	urn:lsid:marinespecies.org:taxname:145520	Fucales	Sargassaceae
*Cystoseiratamariscifolia* (Hudson) Papenfuss, 1950	urn:lsid:marinespecies.org:taxname:145536	Fucales	Sargassaceae
*Desmarestiaaculeata* (Linnaeus) J.V.Lamouroux, 1813	urn:lsid:marinespecies.org:taxname:145307	Desmarestiales	Desmarestiaceae
*Desmarestiadresnayi* J.V.Lamouroux ex Léman, 1819	urn:lsid:marinespecies.org:taxname:145308	Desmarestiales	Desmarestiaceae
*Desmarestialigulata* (Stackhouse) J.V.Lamouroux, 1813	urn:lsid:marinespecies.org:taxname:145309	Desmarestiales	Desmarestiaceae
*Dictyopterispolypodioides* (A.P.De Candolle) J.V.Lamouroux, 1809	urn:lsid:marinespecies.org:taxname:145360	Dictyotales	Dictyotaceae
*Dictyotadichotoma* (Hudson) J.V.Lamouroux, 1809	urn:lsid:marinespecies.org:taxname:145367	Dictyotales	Dictyotaceae
*Ectocarpus* (Lyngbye, 1819)	urn:lsid:marinespecies.org:taxname:144099	Ectocarpales	Ectocarpaceae
*Fucusserratus* (Linnaeus, 1753)	urn:lsid:marinespecies.org:taxname:145546	Fucales	Fucaceae
*Fucusspiralis* (Linnaeus, 1753)	urn:lsid:marinespecies.org:taxname:145547	Fucales	Fucaceae
*Fucusvesiculosus* (Linnaeus, 1753)	urn:lsid:marinespecies.org:taxname:145548	Fucales	Fucaceae
*Halidryssiliquosa* (Linnaeus) Lyngbye, 1819	urn:lsid:marinespecies.org:taxname:145540	Fucales	Sargassaceae
*Halopterisfilicina* (Grateloup) Kützing, 1843	urn:lsid:marinespecies.org:taxname:145906	Sphacelariales	Stypocaulaceae
*Halopterisscoparia* (Linnaeus) Sauvageau, 1904	urn:lsid:marinespecies.org:taxname:145907	Sphacelariales	Stypocaulaceae
*Himanthaliaelongata* (Linnaeus) S.F.Gray, 1821	urn:lsid:marinespecies.org:taxname:145551	Fucales	Himanthaliaceae
*Laminariahyperborea* (Gunnerus) Foslie, 1884	urn:lsid:marinespecies.org:taxname:145725	Laminariales	Laminariaceae
*Laminariaochroleuca* Bachelot de la Pylaie, 1824	urn:lsid:marinespecies.org:taxname:145728	Laminariales	Laminariaceae
*Leathesiamarina* (Lyngbye) Decaisne, 1842	urn:lsid:marinespecies.org:taxname:144953	Ectocarpales	Chordariaceae
*Padinapavonica* (Linnaeus) Thivy, 1960	urn:lsid:marinespecies.org:taxname:145385	Dictyotales	Dictyotaceae
*Pelvetiacanaliculata* (Linnaeus) Decaisne & Thuret, 1845	urn:lsid:marinespecies.org:taxname:145550	Fucales	Fucaceae
*Petaloniafascia* (O.F.Müller) Kuntze, 1898	urn:lsid:marinespecies.org:taxname:145863	Ectocarpales	Scytosiphonaceae
*Phyllariopsispurpurascens* (C.Agardh) E.C.Henry & G.R.South, 1987	urn:lsid:marinespecies.org:taxname:145733	Tilopteridales	Phyllariaceae
*Saccharinalatissima* (Linnaeus) C.E.Lane, C.Mayes, Druehl & G.W.Saunders, 2006	urn:lsid:marinespecies.org:taxname:145730	Laminariales	Laminariaceae
*Saccorhizapolyschides* (Lightfoot) Batters, 1902	urn:lsid:marinespecies.org:taxname:145735	Tilopteridales	Phyllariaceae
*Sargassum* (C.Agardh, 1820)	urn:lsid:marinespecies.org:taxname:144132	Fucales	Sargassaceae
*Sargassummuticum* (Yendo) Fensholt, 1955	urn:lsid:marinespecies.org:taxname:494791	Fucales	Sargassaceae
*Sphacelaria* (Lyngbye, 1818)	urn:lsid:marinespecies.org:taxname:144272	Sphacelariales	Sphacelariaceae
*Taoniaatomaria* (Woodward) J.Agardh, 1848	urn:lsid:marinespecies.org:taxname:145393	Dictyotales	Dictyotaceae
*Treptacanthabaccata* (S.G.Gmelin) Orellana & Sansón, 2019	urn:lsid:marinespecies.org:taxname:145507	Fucales	Sargassaceae
*Treptacanthanodicaulis* (Withering) Orellana & Sansón, 2019	urn:lsid:marinespecies.org:taxname:145526	Fucales	Sargassaceae
*Clionacelata* (Grant, 1826)	urn:lsid:marinespecies.org:taxname:134121	Clionaida	Clionaidae
*Halichondria* (Halichondria) panicea (Pallas, 1766)	urn:lsid:marinespecies.org:taxname:132627	Suberitida	Halichondriidae
*Hymeniacidonperlevis* (Montagu, 1814)	urn:lsid:marinespecies.org:taxname:150223	Suberitida	Halichondriidae
*Acrosoriumciliolatum* (Harvey) (Kylin, 1924)	urn:lsid:marinespecies.org:taxname:295874	Ceramiales	Delesseriaceae
*Ahnfeltiaplicata* (Hudson) (Fries, 1836)	urn:lsid:marinespecies.org:taxname:144422	Ahnfeltiales	Ahnfeltiaceae
*Ahnfeltiopsisdevoniensis* (Greville) P.C.Silva & DeCew, 1992	urn:lsid:marinespecies.org:taxname:145651	Gigartinales	Phyllophoraceae
*Amphiroa* (J.V.Lamouroux, 1812)	urn:lsid:marinespecies.org:taxname:144003	Corallinales	Lithophyllaceae
*Apoglossumruscifolium* (Turner) J.Agardh, 1898	urn:lsid:marinespecies.org:taxname:144737	Ceramiales	Delesseriaceae
*Asparagopsis* (Montagne, 1840)	urn:lsid:marinespecies.org:taxname:295876	Bonnemaisoniales	Bonnemaisoniaceae
*Asparagopsisarmata* (Harvey, 1855)	urn:lsid:marinespecies.org:taxname:144438	Bonnemaisoniales	Bonnemaisoniaceae
*Bonnemaisoniahamifera* (Hariot, 1891)	urn:lsid:marinespecies.org:taxname:144442	Bonnemaisoniales	Bonnemaisoniaceae
*Bornetiasecundiflora* (J.Agardh) Thuret, 1855	urn:lsid:marinespecies.org:taxname:144524	Ceramiales	Ceramiaceae
*Calliblepharisciliata* (Hudson) Kützing, 1843	urn:lsid:marinespecies.org:taxname:145613	Gigartinales	Cystocloniaceae
*Calliblepharisjubata* (Goodenough & Woodward) Kützing, 1843	urn:lsid:marinespecies.org:taxname:145614	Gigartinales	Cystocloniaceae
*Callithamnion* (Lyngbye, 1819)	urn:lsid:marinespecies.org:taxname:143832	Ceramiales	Callithamniaceae
*Callithamniontetragonum* (Withering) S.F.Gray, 1821	urn:lsid:marinespecies.org:taxname:144529	Ceramiales	Callithamniaceae
*Callithamniontetricum* (Dillwyn) S.F.Gray, 1821	urn:lsid:marinespecies.org:taxname:144530	Ceramiales	Callithamniaceae
*Carradorielladenudata* (Dillwyn) A.M.Savoie & G.W.Saunders, 2019	urn:lsid:marinespecies.org:taxname:144623	Ceramiales	Rhodomelaceae
*Catenellacaespitosa* (Withering) L.M.Irvine, 1976	urn:lsid:marinespecies.org:taxname:145605	Gigartinales	Caulacanthaceae
*Caulacanthusustulatus* (Mertens ex Turner) Kützing, 1843	urn:lsid:marinespecies.org:taxname:145606	Gigartinales	Caulacanthaceae
*Ceramiumechionotum* (J.Agardh, 1844)	urn:lsid:marinespecies.org:taxname:144547	Ceramiales	Ceramiaceae
*Ceramiumvirgatum* (Roth, 1797)	urn:lsid:marinespecies.org:taxname:178915	Ceramiales	Ceramiaceae
*Champiaparvula* (C.Agardh) Harvey, 1853	urn:lsid:marinespecies.org:taxname:145804	Rhodymeniales	Champiaceae
*Chondracanthusacicularis* (Roth) Fredericq, 1993	urn:lsid:marinespecies.org:taxname:145623	Gigartinales	Gigartinaceae
*Chondracanthusteedei* (Mertens ex Roth) Kützing, 1843	urn:lsid:marinespecies.org:taxname:162858	Gigartinales	Gigartinaceae
*Chondriacoerulescens* (J.Agardh) Sauvageau, 1897	urn:lsid:marinespecies.org:taxname:1311369	Ceramiales	Rhodomelaceae
*Chondriadasyphylla* (Woodward) C.Agardh, 1817	urn:lsid:marinespecies.org:taxname:144799	Ceramiales	Rhodomelaceae
*Chondruscrispus* (Stackhouse, 1797)	urn:lsid:marinespecies.org:taxname:145625	Gigartinales	Gigartinaceae
*Chylocladiaverticillata* (Lightfoot) Bliding, 1928	urn:lsid:marinespecies.org:taxname:145808	Rhodymeniales	Champiaceae
*Compsothamnionthuioides* (Smith) Nägeli, 1862	urn:lsid:marinespecies.org:taxname:144573	Ceramiales	Ceramiaceae
*Corallina* (Linnaeus, 1758)	urn:lsid:marinespecies.org:taxname:144007	Corallinales	Corallinaceae
*Cryptopleuraramosa* (Hudson) L.Newton, 1931	urn:lsid:marinespecies.org:taxname:144743	Ceramiales	Delesseriaceae
*Delesseriasanguinea* (Hudson) J.V.Lamouroux, 1813	urn:lsid:marinespecies.org:taxname:144744	Ceramiales	Delesseriaceae
*Dilseacarnosa* (Schmidel) Kuntze, 1898	urn:lsid:marinespecies.org:taxname:145222	Gigartinales	Dumontiaceae
*Dumontiacontorta* (S.G.Gmelin) Ruprecht, 1850	urn:lsid:marinespecies.org:taxname:145228	Gigartinales	Dumontiaceae
*Gastrocloniumovatum* (Hudson) Papenfuss, 1944	urn:lsid:marinespecies.org:taxname:145810	Rhodymeniales	Champiaceae
*Gastrocloniumreflexum* (Chauvin) Kützing, 1849	urn:lsid:marinespecies.org:taxname:145811	Rhodymeniales	Champiaceae
*Gelidiumcorneum* (Hudson) J.V.Lamouroux, 1813	urn:lsid:marinespecies.org:taxname:145579	Gelidiales	Gelidiaceae
*Gelidiumpulchellum* (Turner) Kützing, 1868	urn:lsid:marinespecies.org:taxname:145588	Gelidiales	Gelidiaceae
*Gelidiumspinosum* (S.G.Gmelin) P.C.Silva, 1996	urn:lsid:marinespecies.org:taxname:145594	Gelidiales	Gelidiaceae
*Gigartinapistillata* (S.G.Gmelin) Stackhouse, 1809	urn:lsid:marinespecies.org:taxname:145626	Gigartinales	Gigartinaceae
*Gracilariafoliifera* (Forsskål) Børgesen, 1932	urn:lsid:marinespecies.org:taxname:145699	Gracilariales	Gracilariaceae
*Gracilariagracilis* (Stackhouse) Steentoft, L.M.Irvine & Farnham, 1995	urn:lsid:marinespecies.org:taxname:145700	Gracilariales	Gracilariaceae
*Gracilariamultipartita* (Clemente) Harvey, 1846	urn:lsid:marinespecies.org:taxname:145704	Gracilariales	Gracilariaceae
*Grateloupiadoryphora* (Montagne) M.Howe, 1914	urn:lsid:marinespecies.org:taxname:145247	Halymeniales	Halymeniaceae
*Grateloupiafilicina* (J.V.Lamouroux) C.Agardh, 1822	urn:lsid:marinespecies.org:taxname:145248	Halymeniales	Halymeniaceae
*Griffithsia* (C.Agardh, 1817)	urn:lsid:marinespecies.org:taxname:143841	Ceramiales	Wrangeliaceae
*Gymnogongrus* (Martius, 1833)	urn:lsid:marinespecies.org:taxname:144168	Gigartinales	Phyllophoraceae
*Gymnogongruscrenulatus* (Turner) J.Agardh, 1851	urn:lsid:marinespecies.org:taxname:145656	Gigartinales	Phyllophoraceae
*Halopithysincurva* (Hudson) Batters, 1902	urn:lsid:marinespecies.org:taxname:144812	Ceramiales	Rhodomelaceae
*Halurusequisetifolius* (Lightfoot) Kützing, 1843	urn:lsid:marinespecies.org:taxname:146345	Ceramiales	Wrangeliaceae
*Halurusflosculosus* (J.Ellis) Maggs & Hommersand, 1993	urn:lsid:marinespecies.org:taxname:144595	Ceramiales	Wrangeliaceae
*Heterosiphoniaplumosa* (J.Ellis) Batters, 1902	urn:lsid:marinespecies.org:taxname:144732	Ceramiales	Dasyaceae
*Hypneamusciformis* (Wulfen) J.V.Lamouroux, 1813	urn:lsid:marinespecies.org:taxname:145634	Gigartinales	Cystocloniaceae
*Hypoglossumhypoglossoides* (Stackhouse) Collins & Hervey, 1917	urn:lsid:marinespecies.org:taxname:144756	Ceramiales	Delesseriaceae
*Itonoamarginifera* (J.Agardh) Masuda & Guiry, 1995	urn:lsid:marinespecies.org:taxname:145638	Nemastomatales	Nemastomataceae
*Janiarubens* (Linnaeus) J.V.Lamouroux, 1816	urn:lsid:marinespecies.org:taxname:145130	Corallinales	Corallinaceae
*Janiasquamata* (Linnaeus) J.H.Kim, Guiry & H.-G.Choi, 2007	urn:lsid:marinespecies.org:taxname:145114	Corallinales	Corallinaceae
*Laurenciaobtusa* (Hudson) J.V.Lamouroux, 1813	urn:lsid:marinespecies.org:taxname:144827	Ceramiales	Rhodomelaceae
*Leptosiphoniabrodiei* (Dillwyn) A.M.Savoie & G.W.Saunders, 2019	urn:lsid:marinespecies.org:taxname:162854	Ceramiales	Rhodomelaceae
*Lithophyllum* (Philippi, 1837)	urn:lsid:marinespecies.org:taxname:205926	Corallinales	Lithophyllaceae
*Lithophyllumbyssoides* (Lamarck) Foslie, 1900	urn:lsid:marinespecies.org:taxname:145140	Corallinales	Lithophyllaceae
*Lomentariaarticulata* (Hudson) Lyngbye, 1819	urn:lsid:marinespecies.org:taxname:145821	Rhodymeniales	Lomentariaceae
*Lomentariaclavellosa* (Lightfoot ex Turner) Gaillon, 1828	urn:lsid:marinespecies.org:taxname:145825	Rhodymeniales	Lomentariaceae
*Lophosiphoniaobscura* (C.Agardh) Falkenberg, 1897	urn:lsid:marinespecies.org:taxname:146367	Ceramiales	Rhodomelaceae
*Mastocarpusstellatus* (Stackhouse) Guiry, 1984	urn:lsid:marinespecies.org:taxname:145650	Gigartinales	Phyllophoraceae
*Mesophyllumlichenoides* (J.Ellis) Me.Lemoine, 1928	urn:lsid:marinespecies.org:taxname:145188	Hapalidiales	Mesophyllaceae
*Metacallophyllislaciniata* (Hudson) A.Vergés & L.Le Gall, 2017	urn:lsid:marinespecies.org:taxname:145262	Gigartinales	Kallymeniaceae
*Nemalionelminthoides* (Velley) Batters, 1902	urn:lsid:marinespecies.org:taxname:145765	Nemaliales	Nemaliaceae
*Nitophyllumpunctatum* (Stackhouse) Greville, 1830	urn:lsid:marinespecies.org:taxname:144770	Ceramiales	Delesseriaceae
*Osmundeahybrida* (A.P.de Candolle) K.W.Nam, 1994	urn:lsid:marinespecies.org:taxname:144842	Ceramiales	Rhodomelaceae
*Osmundeapinnatifida* (Hudson) Stackhouse, 1809	urn:lsid:marinespecies.org:taxname:144847	Ceramiales	Rhodomelaceae
*Palmariapalmata* (Linnaeus) F.Weber & D.Mohr, 1805	urn:lsid:marinespecies.org:taxname:145771	Palmariales	Palmariaceae
*Peyssonnelia* (Decaisne, 1841)	urn:lsid:marinespecies.org:taxname:144051	Peyssonneliales	Peyssonneliaceae
*Phyllophoracrispa* (Hudson) P.S.Dixon, 1964	urn:lsid:marinespecies.org:taxname:145660	Gigartinales	Phyllophoraceae
*Plocamiumcartilagineum* (Linnaeus) P.S.Dixon, 1967	urn:lsid:marinespecies.org:taxname:145782	Plocamiales	Plocamiaceae
*Polysiphonia* (Greville, 1823)	urn:lsid:marinespecies.org:taxname:143853	Ceramiales	Rhodomelaceae
*Polysiphoniamacrocarpa* (C.Agardh) Sprengel, 1827	urn:lsid:marinespecies.org:taxname:548028	Ceramiales	Rhodomelaceae
*Porphyra* (C.Agardh, 1824)	urn:lsid:marinespecies.org:taxname:143808	Bangiales	Bangiaceae
*Pterocladiellacapillacea* (S.G.Gmelin) Santelices & Hommersand, 1997	urn:lsid:marinespecies.org:taxname:145599	Gelidiales	Pterocladiaceae
*Pterosiphoniacomplanata* (Clemente) Falkenberg, 1897	urn:lsid:marinespecies.org:taxname:146368	Ceramiales	Rhodomelaceae
*Pterothamnioncrispum* (Ducluzeau) Nägeli, 1862	urn:lsid:marinespecies.org:taxname:144682	Ceramiales	Ceramiaceae
*Pterothamnionplumula* (J.Ellis) Nägeli, 1855	urn:lsid:marinespecies.org:taxname:144683	Ceramiales	Ceramiaceae
*Rhodymeniaholmesii* (Ardissone, 1893)	urn:lsid:marinespecies.org:taxname:145853	Rhodymeniales	Rhodymeniaceae
*Rissoellaverruculosa* (Bertoloni) J.Agardh, 1851	urn:lsid:marinespecies.org:taxname:145669	Gigartinales	Rissoellaceae
*Schizymeniadubyi* (Chauvin ex Duby) J.Agardh, 1851	urn:lsid:marinespecies.org:taxname:145673	Nemastomatales	Schizymeniaceae
*Scinaiafurcellata* (Turner) J.Agardh, 1851	urn:lsid:marinespecies.org:taxname:145743	Nemaliales	Scinaiaceae
*Scinaiainterrupta* (A.P.de Candolle) M.J.Wynne, 1989	urn:lsid:marinespecies.org:taxname:239045	Nemaliales	Scinaiaceae
*Sphaerococcuscoronopifolius* (Stackhouse, 1797)	urn:lsid:marinespecies.org:taxname:145908	Gigartinales	Sphaerococcaceae
*Stenogrammainterruptum* (C.Agardh) Montagne, 1846	urn:lsid:marinespecies.org:taxname:145667	Gigartinales	Phyllophoraceae
*Tsengiabairdii* (Farlow) K.C.Fan & Y.P.Fan, 1962	urn:lsid:marinespecies.org:taxname:145649	Halymeniales	Tsengiaceae
*Vertebratafucoides* (Hudson) Kuntze, 1891	urn:lsid:marinespecies.org:taxname:144639	Ceramiales	Rhodomelaceae
*Vertebratanigra* (Hudson) Díaz-Tapia & Maggs, 2017	urn:lsid:marinespecies.org:taxname:144651	Ceramiales	Rhodomelaceae
*Vertebratathuyoides* (Harvey) Kuntze, 1891	urn:lsid:marinespecies.org:taxname:144790	Ceramiales	Rhodomelaceae
*Xiphosiphoniapennata* (C.Agardh) Savoie & G.W.Saunders, 2016	urn:lsid:marinespecies.org:taxname:144852	Ceramiales	Rhodomelaceae

**Table 3.** Number of taxa identified per Phylum in each location. A - Cnidaria; B - Echinodermata; C - Arthropoda; D - Chordata; E - Annelida; F - Mollusca; G - Porifera; H - Rhodophyta; I - Ochrophyta; J - Chlorophyta; K - Ascomycota; L - Bryozoa

**Location**	**A**	**B**	**C**	**D**	**E**	**F**	**G**	**H**	**I**	**J**	**K**	**L**	**Total**
Moledo do Minho	3	3	5	2	2	11	1	33	12	2	2	0	76
Vila Praia de Âncora	3	3	8	1	2	14	1	40	13	2	2	0	89
Afife	3	3	11	2	1	15	1	32	12	2	2	1	85
Montedor	6	5	15	5	4	26	3	41	13	2	1	0	121
Forte da Vigia	0	0	0	0	0	0	0	32	13	3	1	0	49
Praia Norte	1	2	3	1	1	11	0	16	7	1	1	0	44
Amorosa	4	2	7	3	1	15	2	29	15	2	0	1	81
Mindelo	4	2	6	0	2	8	1	34	11	2	2	0	72
Vila Chã	2	3	3	1	1	11	0	37	12	2	1	0	73
Labruje	2	3	5	2	2	12	1	21	9	2	0	1	60
Angeiras	3	1	7	3	1	14	1	24	9	2	0	0	65
Cabo do Mundo	5	3	4	0	1	13	0	23	6	2	0	1	58
Homem do Leme	4	3	7	2	1	13	1	27	4	2	1	1	66
Valadares	2	2	2	0	1	8	1	26	3	2	0	0	47
Miramar	2	0	3	0	1	10	1	12	4	2	0	0	35
Aguda	4	1	7	2	1	9	1	35	5	2	0	1	68
Buarcos	2	2	4	1	1	8	1	26	4	3	1	0	53
Nazaré	1	2	10	1	3	19	3	84	13	7	1	0	144
São Martinho do Porto	2	2	4	1	1	13	2	26	7	2	1	0	61
Baleal	3	1	7	1	1	11	1	43	14	5	1	0	88
Papoa	2	1	4	0	0	9	1	20	6	4	2	0	49
São Bernardino	2	2	4	1	1	11	1	14	6	2	1	0	45
Santa Cruz	2	0	3	1	2	12	0	16	4	3	1	0	44
São Lourenço	6	2	6	1	2	12	1	23	9	2	2	0	66
Ericeira	2	2	4	2	1	9	1	30	7	2	0	0	60
São Julião	1	0	3	2	2	11	0	15	3	3	0	0	40
Magoito	3	1	5	0	2	9	1	20	5	2	0	0	48
Adraga	1	0	4	0	1	7	1	11	4	2	0	0	31
Abano	2	0	5	1	2	10	1	9	0	1	1	0	32
Cabo raso	4	1	5	2	2	11	1	37	6	4	2	0	75
Avencas	2	3	8	3	1	15	1	26	7	3	0	0	69
Cabo Espichel	2	3	2	0	0	8	0	11	5	4	0	0	35
Portinho da Arrábida	2	0	3	1	2	9	1	24	8	4	1	0	55
São Torpes	3	1	7	3	2	11	0	29	9	3	1	0	69
Oliveirinha	2	1	4	0	2	8	0	29	13	6	0	0	65
Queimado	4	1	8	3	2	13	1	23	10	4	1	0	70
Vila Nova de Milfontes	1	0	4	0	1	9	0	28	9	3	1	0	56
Zambujeira do Mar	3	2	5	0	1	11	2	37	9	3	1	0	74
Vale dos Homens	6	2	6	2	3	12	1	27	6	3	0	0	68
Monte Clérigo	3	1	4	0	2	13	1	33	12	3	1	0	73
Arrifana	8	2	7	3	2	12	1	25	5	2	1	1	69
Castelejo	4	1	5	2	0	9	2	38	8	3	1	1	74
Martinhal	3	6	8	4	1	13	0	19	8	5	2	0	69
Ingrina	1	2	4	0	2	15	0	32	9	4	0	1	70
Praia da Luz	2	2	4	0	2	11	2	23	9	4	1	0	60
Porto de Mós	1	1	8	6	2	14	0	17	9	3	1	0	62
Dona Ana	1	2	5	0	1	8	0	24	6	5	0	0	52
Castelo	2	1	6	1	2	9	1	26	9	4	0	0	61
Olhos de Água	5	1	8	0	3	12	0	17	6	4	1	1	58
